# Follow-up practices for high-grade extremity Osteosarcoma

**DOI:** 10.1186/s12885-016-2333-y

**Published:** 2016-05-06

**Authors:** Christian Rothermundt, Beatrice M. Seddon, Palma Dileo, Sandra J. Strauss, Joanne Coleman, Timothy W. Briggs, Sarah R. Haile, Jeremy S. Whelan

**Affiliations:** Division of Oncology/Haematology, Kantonsspital St. Gallen, Rorschacherstrasse 95, 9007 St. Gallen, Switzerland; London Sarcoma Service, University College London Hospitals, London, NW1 2BU UK; Department of Orthopaedic Surgery, Royal National Orthopaedic Hospital, Middlesex, HA7 4LP UK; Epidemiology, Biostatistics and Prevention Institute, University of Zurich, Hirschengraben 84, 8001 Zürich, Switzerland

**Keywords:** Osteosarcoma, Follow-up, Imaging

## Abstract

**Background:**

The optimal conduct of follow-up (FU) of patients with osteosarcoma is uncertain. In the absence of any formal validation of optimal timing and method of surveillance, guidance is provided by oncology societies’ recommendations. FU is designed to detect either local recurrence or metastatic disease at a time when early treatment is still possible and might be effective.

**Methods:**

We performed a retrospective analysis of 101 patients with high-grade extremity osteosarcoma in a single centre. Chest x-ray (CXR) was used as routine surveillance method; however patients with initial lung metastases or previous suspicious findings had computed tomography (CT) scans.

**Results:**

With a median FU time of 30.7 months 34 patients relapsed. Relapse–free survival after 5 years was 61 % (CI 52 %; 73 %), late relapses occurred in only two patients between 2 and 5 years of FU.

Twenty-five of the 34 relapses were detected at routine FU appointments. All 8 local recurrences were noted clinically. Twenty-two patients had metastases confined to the lungs, either detected on CXR or CT. Thirty-two percent of patients with lung metastases only were salvaged successfully.

**Conclusions:**

Routine FU in high-grade osteosarcoma results in clinical detection of local relapse, and detection of lung metastases by CXR at a time when metastatectomy is possible. The optimal time interval for FU appointments is not known, however we recommend more frequent surveillance visits during the two years after treatment. We hypothesize that routine CT scans are not required and propose CXR for detection of lung metastases.

**Electronic supplementary material:**

The online version of this article (doi:10.1186/s12885-016-2333-y) contains supplementary material, which is available to authorized users.

## Background

High-grade osteosarcoma is the most common primary bone tumour. It is a disease of childhood, adolescence and young adulthood [1]. Osteosarcoma survival rates for children and adolescents in Europe showed marked improvement up to the 1980s [2] and many osteosarcoma patients are cured by multi-agent chemotherapy and surgery [3]. However, a significant number of patients who are rendered free of disease by initial chemotherapy and surgery develop disease relapse. The opportunity to achieve a second complete remission by surgical resection is essential for survival [4].

FU time intervals, duration and investigations vary after treatment for high-grade extremity osteosarcoma [5, 6]. FU is designed to detect either local recurrence or metastatic disease at a time when early treatment is still possible and might be effective [7]. One prospective randomized trial on surveillance intensity in extremity sarcoma (soft tissue and bone) was recently reported [8]. Nevertheless, the optimal frequency of FU and the best radiologic method of lung surveillance continue to be unknown. Radiation exposure is a concern especially in young patients and should be balanced with the potential benefits of early detection of relapse [9, 10].

## Methods

This is a retrospective analysis of 101 consecutive patients with high-grade extremity osteosarcoma who presented to a single institution for treatment and/or FU from 2003 to 2009. Routine FU consisted of clinical examination, CXR and plain films of primary site 2-monthly year 1, 3-monthly year 2 and 3, and 6-monthly year 4 and 5. Confirmatory CT scans were performed in all patients with suspicious findings. Routine CT scans were performed at the end of treatment, when suspicious finding had been observed previously and in a patient with resected lung metastases. Patients were routinely seen and assessed by the treating oncologist or the surgeon. Patient and tumour data were collected from hospital records. Data collection was in accordance with local ethical standards [11]. Survival rates were computed using the method of Kaplan and Meier [12, 13]. As 50 % survival was not reached, 1, 2, and 5 year survival rates with 95 % confidence intervals are presented. Event rates by site of relapse were examined using cumulative incidence functions for survival data with competing risks [14]. No hypothesis testing has been performed. All analysis was performed in the R programming language (version 3.2.2) [15].

## Results

Median age at surgery was 18.7 years (range (4.7, 66.3)). The primary tumour was located in the lower extremity in 87 patients, and in the upper extremity in 14 patients. Ninety-three patients had localized disease, 5 patients had lung metastases at diagnosis, 2 patients a single bone and 1 patient a lymph node metastasis. All patients underwent surgery, 100 patients received chemotherapy, 92 pre- and postoperatively. Standard chemotherapy consisted of high-dose methotrexate, doxorubicin and cisplatin (MAP) [16]. Eight patients had radiotherapy. With a median FU time of 30.7 months (range (2.2 months, 101.6 months)), 34 patients relapsed.

**Fig. 1:**
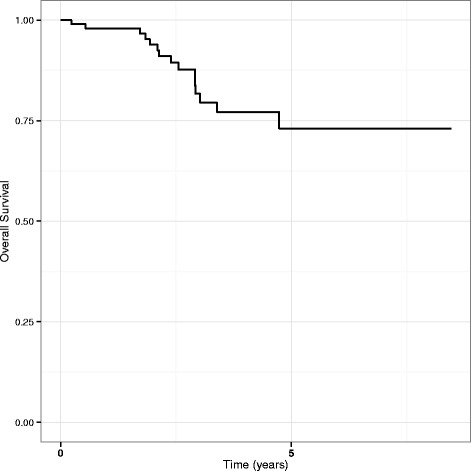
Overall survival

Five of the 8 patients with initial metastases (60 %), and 29 patients with initially localised disease relapsed (31 %). Of 34 patients with a relapse, 15 died (44 %). Overall survival (OS) estimates for all patients at 1, 2, and 5 years are 98, 94 and 73 %, respectively (Fig. 1).

**Fig. 2:**
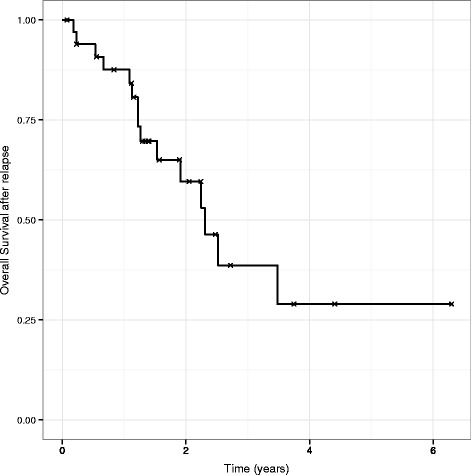
Overall survival after relapse

Survival from time of relapse estimates at 1, 2, and 5 years are 88, 60 and 29 %, respectively (Fig. 2).

Twenty-five of the 34 relapses were detected at routine FU appointments. All 8 local recurrences were noted clinically, seven by the patients and one by the physician. Signs or symptoms of local recurrence were masses or swelling in 7 patients and pain in 1 patient, respectively. Half of the local relapses were detected or reported at routine FU, the other half outside scheduled appointments. The four patients with local relapse only were treated with curative intent and three patients were salvaged. However, three of the four patients with local relapse and synchronous metastases died, one patient was alive but not cured.

Twenty-two patients had metastases confined to the lungs (Table 1). Three patients were symptomatic at relapse with a pleural effusion, cough, and a pulmonary embolism. In 10 patients routine CXR revealed pulmonary metastases. In 9 patients lung metastases were detected on planned CT: 4 on end of treatment scans, 3 with suspicious findings during or at end of treatment, 1 had lung metastases resected and was followed by CT, in one patient the indication for a CT scan is unclear.

**Table 1:** Sites of relapse

Site	n	% of relapses
Local	4	12
Local and lung	3	9
Local and bone	1	3
Lung	22	65
Lung and bone	3	9
Bone	1	3

Nine of the 10 patients with CXR-detected lung metastases were treated with curative intent by surgery only (*n* = 4) or surgery and chemotherapy (*n* = 5) and 5 remain relapse-free. Among the 9 patients with CT detected lung metastases 6 were treated with curative intent and 2 remain relapse-free. In summary, 32 % of patients with lung metastases only were salvaged successfully. The higher rate of salvaged patients after CXR-detected compared to CT-detected lung metastases is probably attributable to the fact that the baseline risk of relapse was higher in the patient group followed with chest CT scans.

**Fig. 3:**
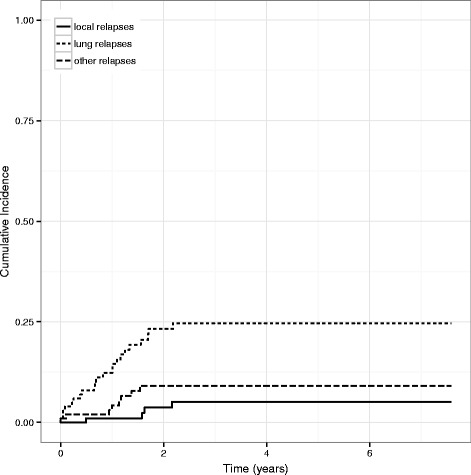
Cumulative incidence of relapse

Most relapses occurred within the first two years after end-of treatment and only two patients experienced a relapse between years 2 to 5. Relapse-free survival estimates at 1, 2, and 5 years are 83, 64 and 61 %, respectively (Fig. 3).

In 101 patients, there were a total of 1’004 appointments without detection of relapse, corresponding with costs of Great Britain Pound (GBP) 151’604 (outpatient appointment (OPA) at University College London Hospitals (UCH) + CXR + plain films of primary site) to GBP 287’144 (OPA at UCH + CT + plain films of primary site). Median number of visits without relapse detected was 8 (0–21), with costs of either GBP 1’210 (0 – 3’170) or 2’290 (0 – 6’010). It is notable that none of the relapses were detected after 26 months, even after a potential FU to 91 months.

## Discussion

In this small retrospective analysis of 101 patients with high-grade extremity osteosarcoma the median time to relapse and the sites of relapse are similar to larger series [3]. It confirms that the lungs are the main site of metastases from osteosarcoma [17]. Nineteen of the 34 patients (56 %) who experienced relapse were still alive in our analysis after a median FU time of 30.7 months (range (2.2 months, 101.6 months)); however FU was much shorter compared to a previously reported series of 110 relapsed osteosarcoma patients and a survival rate of 16.4 % [4] after a median FU of 13.7 years (range (4.6 years, 33.5 years)). In another retrospective analysis the projected 5-year post relapse survival (PRS) rate was 28 %. Patients who had complete surgery of recurrence had a 5-year PRS of 39 %, whereas for those who did not have complete surgery, PRS was 0 % at 3 years [18]. The importance of complete resection was also demonstrated in two other retrospective reviews [19, 20]. Combination chemotherapy may contribute to a modest improvement in outcome for relapsed patients [3].

One limitation of this retrospective analysis is that we cannot present data on histological response to preoperative chemotherapy, which is an independent prognostic parameter for relapse and survival [21]. In European and American Osteosarcoma Study Group (EURAMOS)-1 50 % of osteosarcoma patients obtained a good response to preoperative MAP chemotherapy, and 50 % had a poor response [22]. After a median FU of 44 months 174 events were reported in 716 patients with good histological response [23], whereas 300 events were reported in 618 patients with a poor histological response after a median FU of 54 months, respectively [24]. Due to the differences in frequency of events, FU might ideally be adjusted to the risk of relapse according to histological response.

We do not report on results of blood tests, which are routinely performed during FU. We think blood tests are unhelpful in detecting relapses. However, blood tests might be useful to detect late organ toxicity following intensive chemotherapy.

The only randomised trial of FU strategies in sarcoma patients [8] compared chest imaging modalities (CXR and CT scans) and frequency of FU (3-monthly and 6-monthly visits) in 359 bone and 151 soft tissue extremity sarcomas. CXR was non-inferior as compared with CT scans (3-year OS 67 and 66 %, respectively; disease-free survival 54 and 49 %, respectively). However the trial could not conclusively demonstrate non-inferiority for less frequent FU visits.

Most relapses in our series occurred within the first two years after end-of treatment and when patients are seen every two to three months, we therefore doubt that more frequent visits and scans would have altered the course of disease. However, we cannot exclude this nor that in the two patients, who experienced a relapse between years 2 to 5, earlier detection of relapse would have made treatment easier and more successful.

The question remains whether early relapse is a sign of more aggressive disease and early detection can substantially change the course of the disease and outcome.

We previously reported data from a retrospective analysis of patients with extremity soft tissue sarcoma and showed that routine CXR in FU can detect lung metastases suitable for surgical resection. Local relapse of soft tissue sarcoma is almost always detected by patients or physicians, and routine scanning of the primary site is of doubtful benefit [25]. In line with this data, we now report a clinical detection rate of 100 % (8/8) for local relapse in high-grade extremity osteosarcoma. X-rays of the primary tumour site during surveillance of osteosarcoma may be useful to inform on reconstructive results and prosthesis function [26, 27], but are of little relevance for detection of local relapse.

CXR detected pulmonary metastases in 10/22 patients, 9 patients underwent complete resection of lung metastases, 5 of whom were relapse-free at last FU (2 after surgery and 3 after surgery and chemotherapy). Patients with CT detected pulmonary metastases were either assessed at end of treatment (*n* = 4), due to suspicious findings during or at end of treatment (*n* = 3), or after lung metastases resection (*n* = 1). In one patient the reason for a CT scan remains unclear. Among patients with CT detected pulmonary metastases 2 remain relapse-free at last FU. We postulate that CXR is sufficient for routine scanning of the lungs, however we would advocate CT scans in patients with suspicious findings on CXR or previous abnormalities, which require following.

## Conclusion

Routine FU with clinical examination by patient and physician supplemented by CXR in high-grade extremity osteosarcoma results in clinical detection of local relapse, and detection of lung metastases at a time when metastatectomy is possible. The optimal time interval for FU appointments is not known. The higher relapse-rate during the first two years of FU suggest more frequent examination, however this has not been shown to have impact on outcome. We hypothesize that 2- or 3-monthly FU during the first 2 years after curative treatment for high-grade extremity osteosarcoma with CXR is feasible and may be sufficient in patients who had no previous abnormalities and remain asymptomatic.

### Ethics approval and consent to participate

No individual consent has been obtained from patients. Only anonymized data from the University College London Hospital database was used. This analysis is formally counted as service evaluation. Therefore, no formal ethics committee approval is necessary according to local standards [11].

### Consent for publication

Not applicable.

### Availability of data and materials

The dataset supporting the conclusions of this article is included within the article and its Additional file 1.

## Additional file

Additional file 1: Bone Sarcoma Data Set. (CSV 6 kb)

## Abbreviations

CT: computed tomography
CXR: chest x-ray
EURAMOS: European and American Osteosarcoma Study Group
FU: follow-up
GBP: Great Britain pound
MAP: high-dose methotrexate, doxorubicin and cisplatin
OPA: outpatient appointment
OS: overall survival
PRS: post relapse survival
UCH: University College London Hospitals
